# *Vaccinium exiguum* (Ericaceae, Vaccinieae), a new species from the ultramafic summit of Mt. Victoria, Palawan Island, Philippines

**DOI:** 10.3897/phytokeys.179.68323

**Published:** 2021-07-21

**Authors:** Maverick N. Tamayo, Rene Alfred Anton Bustamante, Peter W. Fritsch

**Affiliations:** 1 Department of Biology, College of Science, University of the Philippines Baguio, 2600, Baguio City, Philippines University of the Philippines Baguio Philippines; 2 Philippine Taxonomic Initiative Inc., Botanica Building, El Nido, 5313, Palawan, Philippines Philippine Taxonomic Initiative Inc. El Nido Philippines; 3 Botanical Research Institute of Texas, 1700 University Drive, Fort Worth, 76107, Texas, USA Botanical Research Institute of Texas Fort Worth United States of America

**Keywords:** Ericales, endemic species, Malesia, sect. *Bracteata*, Vaccinioideae

## Abstract

*Vaccinium exiguum* from the ultramafic summit of Mt. Victoria, Palawan Island, Philippines is here described as a new species of Ericaceae. It closely resembles *V. hamiguitanense* but is distinct by having much shorter petioles and leaves, longer and glabrous calyx lobes with serrate lobe margins, a larger corolla with deeper sulcations, and longer stamens with spurs oriented laterally. *Vaccinium exiguum* represents the third *Vaccinium* species found on the Island of Palawan and 36^th^ in the Philippines.

## Introduction

The Island of Palawan is situated on the south-western side of the Philippine Archipelago and is bordered by the West Philippine Sea in the north and the Sulu Sea in the south. Palawan is a biodiverse area, regarded both biogeographically and geologically as a portion of the Sunda Shelf, with many of its species shared with Borneo (Dickerson 1928; Heaney 1986; Voris 2000; Esselstyn et al. 2004). The long and complex geohistory of the island which started during the mid-Oligocene, as well as its highly variable elevation and climate, has promoted high rates of speciation and endemism (Anacker 2011; Galey et al. 2017). In particular, the extensive areas of ultramafic substrates have resulted in the evolution of many endemic plant species in Palawan (e.g. Robinson et al. 2009, 2016; Malabrigo 2020; Quakenbush et al. 2020; Tandang et al. 2020) and it is to be expected that more species await discovery in these ultramafic regions as they are further explored and studied.

The tropical species of *Vaccinium* L. are predominantly montane inhabitants with a high degree of endemicity (Argent 2018). The genus is the most species-rich of the Philippine genera of the family and is currently represented by 35 species, 32 of which are endemic to the country (Argent 2008; Pelser et al. 2011 onwards). The highly regarded taxonomic treatments by Copeland (1930) and Sleumer (1966–1967) are the most comprehensive, thus far, for Philippine *Vaccinium*. However, gaps in our knowledge of Philippine *Vaccinium* remain, especially in various species complexes (e.g. *V. caudatum* Warb./*V. benguetense* S.Vidal) and many character ambiguities used for the treatments require clarification and resolution. After Sleumer’s work (1966–1967), three species have been added to the list, viz. *V. cebuense* Salares and Pelser, *V. hamiguitanense* P.W.Fritsch, and *V. oscarlopezianum* Co (Co et al. 2002; Salares et al. 2018; Fritsch et al. 2020).

**Figure 1.**
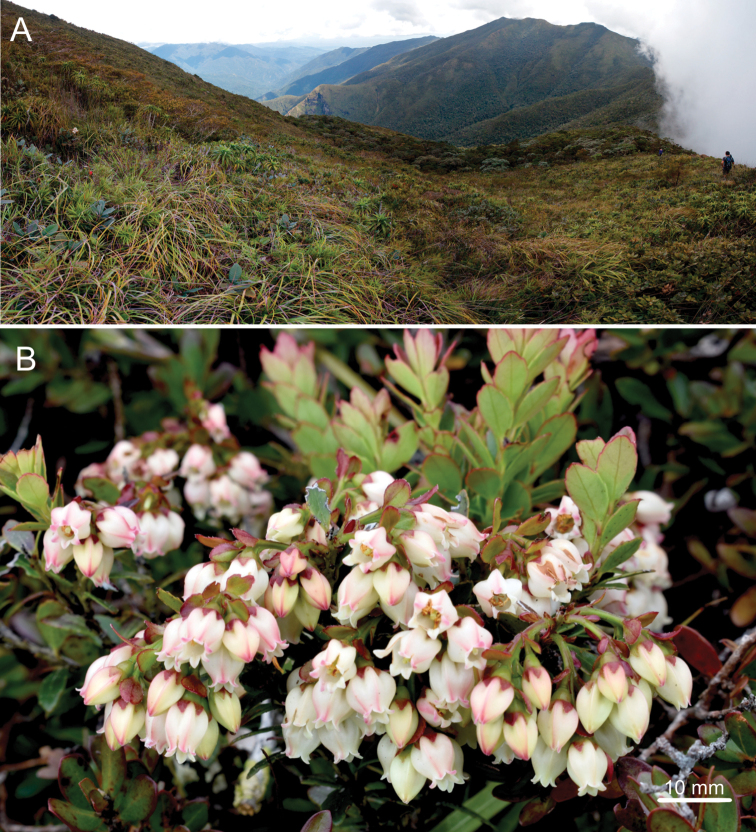
*Vaccinium exiguum***A** ultramafic summit of Mt. Victoria **B** flowering branchlets *in situ*. Photo **A** by Alastair Robinson. Photo **B** by Rene Alfred Anton Bustamante.

During fieldwork on Mt. Victoria, Palawan Island in February 2021, author Bustamante documented a species of *Vaccinium* that grows at the ultramafic summit of the mountain (Fig. 1A) and closely resembles the recently described *V. hamiguitanense* from Mt. Hamiguitan, Mindanao Island. However, the inflorescence of this species differs from that of *V. hamiguitanense* in shape. After detailed morphological examination, it was confirmed that the specimen possesses distinguishing characters demonstrating its status as a species new to science, which we describe here under a biological species concept (Mayr 2000). Our discovery increases the number of species of *Vaccinium* in the Philippines to 36 and increases the number of known *Vaccinium* species from Palawan Island to three. Photographs and an illustration of the new species are also provided.

## Materials and methods

The description was based on *in-situ* observations, herbarium specimens, and spirit samples preserved in Copenhagen solution. Microscopic details were described with the aid of a Swift SM100 stereo zoom microscope (30× magnification). Relevant literature and taxonomic keys (i.e. Copeland 1930; Sleumer 1966–1967; Argent 2018) were also consulted.

## Taxonomy

### Vaccinium exiguum

Taxon classificationPlantaeEricalesEricaceae

M.N.Tamayo, R.Bustam. & P.W.Fritsch sp. nov.

3A8D62B5-3C2E-51EE-AAF6-7A8D800933FD

urn:lsid:ipni.org:names:77218415-1

Figs 1B , 2 , 3

#### Type.

Philippines, Palawan Island (= Palawan Province), Municipality of Narra, Mt. Victoria, 9°21'56"N, 118°20'02"E, exposed ultramafic summit, ca. 1700 m elevation, 27 February 2021, *PTI-8 by R. Bustamante* (holotype: PNH!; isotypes: PPC!; BRIT!).

#### Diagnosis.

*Vaccinium exiguum* closely resembles *V. hamiguitanense*, but is distinct by having shorter petioles (0.5–1.0 mm vs. 1.2–1.8 mm), shorter leaves (4.9–9.3 mm vs. 8.8–16.0 mm), longer calyx lobes (1.8–2.0 mm vs. 1.1–1.3 mm) that are glabrous (vs. merely ciliate) with serrate calyx lobe margins (vs. entire), a larger corolla (6.0–7.0 × 4.0–5.0 mm vs. 4.6–5.0 × ca. 2.6 mm) with deeper (vs. shallow) sulcations, and longer stamens (3.5–4.0 mm vs. 2.8–3.5 mm) with spurs oriented laterally (vs. slightly upcurved and oriented ± apically). The calyx lobes of *V. exiguum* are unique amongst Philippine species in that they are nearly as long as or longer than the hypanthium. Unlike *V. hamiguitanense*, *V. exiguum* has a corolla that appears distinctly star-shaped in cross section because of its deep sulcations.

#### Description.

***Habit*** shrubs, terrestrial, evergreen, 0.3–1.5 m tall, densely branched. ***Branchlets*** when young with finely translucent erect, straight trichomes ca. 0.2 mm long, at maturity brown, slightly compressed and often ridged, 0.5–1.5 mm wide, not lenticellate, outer surface brownish; perennating buds compressed-ovoid, 0.5–0.8 mm long, with several obscurely overlapping scales. ***Leaves*** persistent on older branchlets, densely crowded, spirally and evenly arranged; petiole green with a tinge of red, 0.5–1.0 × 0.4–0.8 mm, nearly as long as wide, with translucent, erect, straight trichomes ca. 0.1 mm long, in cross section abaxially rounded, adaxially nearly flat; leaf blade elliptic or oblong, larger leaves on each branchlet 4.9–9.3 × 3.0–6.0 mm, coriaceous, both surfaces greenish red when young, glabrous, except occasionally puberulent at base, smooth, abaxial surface without punctae, green and glossy, light brown *in sicco*, adaxial surface green and nitid, dark brown *in sicco*, base cuneate, margin with 4 or 5 impressed ± evenly distributed crenations per side with occasional minute translucent lanceolate glands on crenations, thinly recurved, apex obtuse to rounded, the very tip with a gland, marginal glands 3 or 4 per side, scattered along length of margin, ca. 0.1 mm diameter, midvein slightly raised abaxially, flattened adaxially or nearly so, secondary veins 2 to 4 on each side of midvein with first pair arising from base and remainder along midvein, arc-ascending, slightly raised or obscure abaxially, obscure adaxially, tertiary veins faintly evident or obscure. ***Inflorescences*** pseudo-terminal or terminal, racemose, ca. 1.5 cm long at anthesis, developing beyond confines of perennating bud, densely flowered, 3 to 8 per axil, (3 to)5 or 6-flowered; rachis green, puberulent, slightly ridged with translucent trichomes ca. 0.1 mm long; bracts subtending pedicels, foliaceous, greenish, brown *in sicco*, ovate to elliptic, planar or occasionally cucullate, 3.0–8.0 × 4.0–7.5 mm, coriaceous, glabrous, margin crenulate or serrulate with 4 or 5 (or 6) impressed crenations per side, with minute translucent lanceolate glands on crenations, apex obtuse or rounded. ***Flowers*** articulated at junction with pedicel, 6.0–7.0 mm long. ***Pedicel*** nodding, 2.0–5.0 × 0.8–0.9 mm at anthesis, white-puberulent; bracteoles persistent, 2, borne at base of pedicel, margin with minute translucent lanceolate glands on crenations, linear-lanceolate to oblong or nearly so, 1.0–1.2 × 0.3–0.5 mm long, glabrous, margin serrate, apex sharply acute. ***Hypanthium*** green, glossy, cupuliform, 1.4–1.8 × 0.9–1.0 mm, glabrous; calyx limb 0.7–1.0 mm long, glabrous; calyx lobes broadly triangular, 1.8–2.0 mm long, glabrous both sides, margin serrate, with minute translucent lanceolate glands on crenations, apex acute, without sessile and terminal gland. ***Corolla*** in bud closed, broadly urceolate and strongly 5- to 7-ribbed (sulcations) along the petal midveins, pale green, at anthesis strongly 5- to 7- ribbed, white with tinge of pale red or pink near and on lobes, 6.0–7.0 × 4.0–5.0 mm, glabrous inside and outside; corolla lobes 5 to 7, ca. 1.0 × 1.0 mm, apex acute or obtuse. ***Stamens*** 8 to 10, monomorphic, free from each other, 3.5–4.0 mm long; filaments straight, 1.9–2.0 mm long, white-pubescent mainly at base, trichomes ca. 0.1 mm long (shorter distally); anthers 1.5–2.1 mm long, cells 1.2–1.3 mm long, echinulate, tubules parallel, broadly cylindrical, 0.8–1.0 mm long, slightly narrower than cells, opening by oblique ventrally oriented apical pores, pore apex rounded, spurs present, minute, borne ± midway along anther, laterally oriented, 0.15–0.20 mm long. ***Ovary*** 5 or 6 (or 7)-locular, but appearing pseudo-10- to 12- (to 14-) locular with false partitions extending 0.20–0.25 mm from inner wall; ovules in two columns per locule, each column separated by false partitions; disk circular with prominent ridges on margin, ca. 2.0 mm in diameter, glabrous; style not exserted from corolla, 4.0–5.0 mm long, glabrous. Fruit not observed.

**Figure 2.**
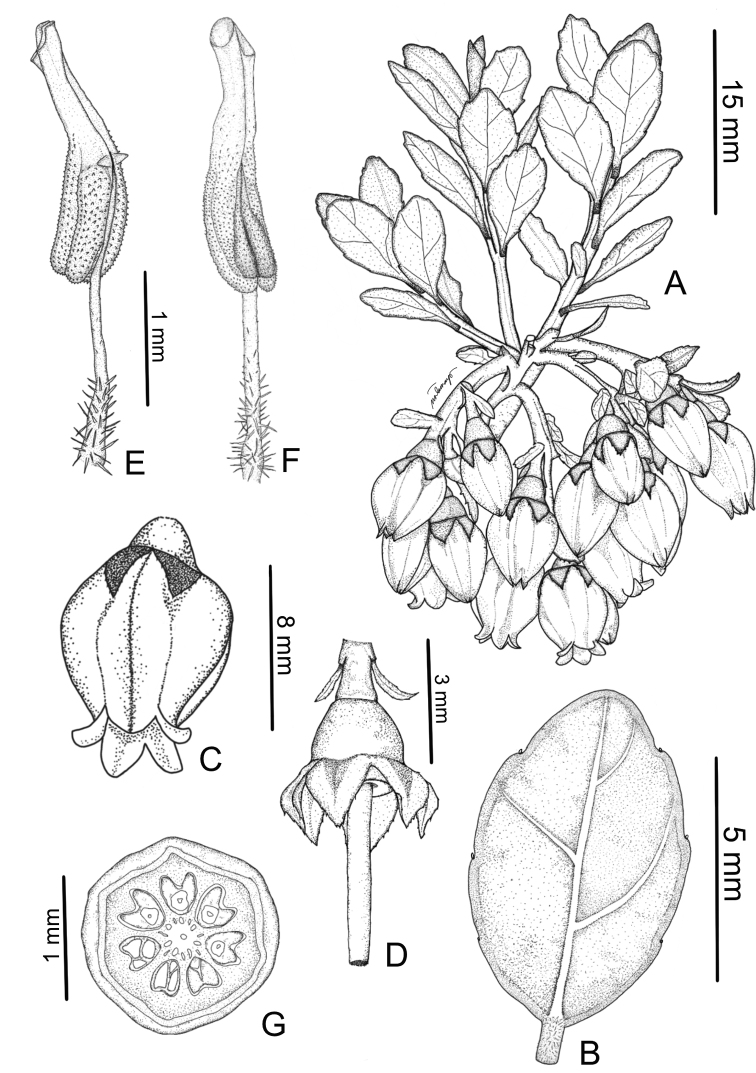
*Vaccinium exiguum***A** flowering branchlet **B** leaf, abaxial view **C** lateral view of flower showing ribbed corolla **D** distal portion of pedicel, as well as hypanthium, calyx lobes and style **E** stamen in oblique-lateral view showing spurs **F** stamen in ventral view **G** cross section of ovary showing seven locules. Illustrated by Maverick N. Tamayo.

**Figure 3.**
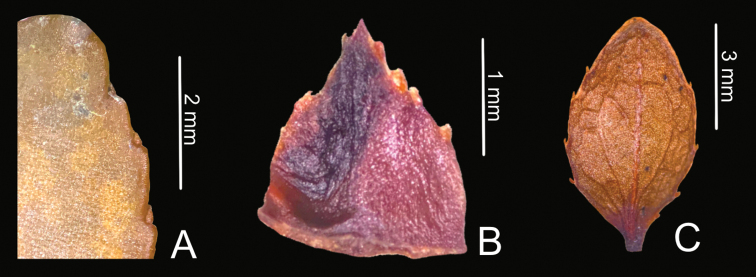
Translucent glands of *Vaccinium exiguum***A** leaf margin **B** calyx lobe **C** bract. Photos by Maverick N. Tamayo.

#### Etymology.

The epithet “*exiguum*” refers to the overall small stature and leaf morphology of the new species.

#### Distribution and habitat.

*Vaccinium exiguum* is currently known from a single individual from a single location at the exposed ultramafic forest summit of Mt. Victoria, Palawan Island, Philippines at ca. 1700 m elevation.

#### Conservation status.

Only a single flowering plant was documented from a single location at the summit of Mt. Victoria, Palawan. Other summits within the mountain range are similar to the type locality in elevation, but these have yet to be explored. As such, we propose the conservation threat status Data Deficient (DD) (IUCN Standards and Petitions Committee 2019) due to the scarcity of data with which to confidently assess the species against the IUCN guidelines.

Many species are endemic to Mt. Victoria (Robinson et al. 2009, 2016). Though harboring high biodiversity, Mt. Victoria is unfortunately not legislated as a protected area (PA). Mining activities within the area are considered a significant threat that poses the risk of habitat and forest degradation. Thus, the lack of legal protection is the greatest threat to this species and to the biodiversity of the area.

#### Phenology.

The new species was observed flowering during the month of February.

## Discussion

*Vaccinium exiguum* belongs to section Bracteata Nakai (Nakai and Koidzumi 1927) sensu Sleumer (Sleumer 1966–1967) as exhibited by its well-developed and often many-flowered racemes, corolla consisting of a single homogenous layer, and the absence of a membranaceous wing at the sinuses and anthers that open by short introrse slits or terminal pores (Co et al. 2002; Salares et al. 2018; Fritsch et al. 2020).

In the key of Sleumer (1996–1967) of the Malesian Vaccinium section Bracteata, *V. exiguum* best keys to *V. gitingense* Elmer (endemic to the Philippines). However, the new species differs from *V. gitingense* by having shorter petioles (0.5–1.0 mm vs. ca. 2.0 mm), smaller leaf blades (4.9–9.3 × 3.0–6.0 mm vs. 15–35 × 8–15 mm), glabrous calyx lobes (vs. finely ciliate), a non-glaucous and glabrous corolla (vs. glaucous and occasionally bears few hairs), and longer filaments (1.9–2.0 mm vs. ca. 1.5 mm) (Elmer 1912). In the key to the Bornean species of *Vaccinium* (Argent 2018), the species best keys to V. coriaceum var. stapfianum (Sleumer) Argent (restricted to Mt. Kinabalu, Malaysia); however, *Vaccinium exiguum* differs by having shorter leaf blades (4.9–9.3 mm vs. 6.0–13.0 mm), a crenate leaf margin (vs. entire), longer calyx lobes (1.8–2.0 mm vs. ca. 1.3 mm) that are triangular (vs. ovate), and a corolla with deep sulcations (vs. no sulcations). Likewise, the presence of translucent lanceolate glands on the crenations along the margins of the leaves, bracts, bracteoles, and calyx lobes of *V. exiguum* (Fig. 3) is a character shared by the three species.

Although the species exhibits morphological similarities with *V. hamiguitanense*, the flowers of *V. exiguum* are unique amongst all other Philippine *Vaccinium* in having a broadly urceolate and strongly 5- to 7-ribbed corolla and calyx lobe margins that are serrate. *Vaccinium exiguum* also possesses the smallest leaves amongst the Philippine *Vaccinium* species with a size range near *V. hamiguitanense* and *V. microphyllum* Reinw. The latter species is easily distinguished from *V. exiguum* by its axillary solitary flowers (vs. terminal multi-flowered inflorescences). In addition to the characters distinguishing *V. exiguum* from *V. hamiguitanense* as specified in the diagnosis, the two species have distinct geographical ranges.

Currently, two species of *Vaccinium* are recorded in Palawan: *V. brachytrichum* Sleumer and *V. palawanense* Merr. The new species can be easily differentiated from *V. brachytrichum* by bearing fewer flowers per inflorescence [(3 to)5 or 6 vs. 5 to 8)], longer anthers (1.5–2.1 mm vs. ca. 1 mm), and a glabrous (vs. pubescent) hypanthium. It differs from *V. palawanense* in the short and densely flowered inflorescences that are much shorter than the leaves (vs. flowers in racemes about as long as the leaves), a shorter pedicel (2.0–5.0 mm vs. 5.0–7.0 mm), and a 5- or 6-(or 7-) locular ovary (vs. 5) (Merrill 1908; Sleumer 1961). Moreover, the strongly ribbed corolla and the small leaves with crenate margins are also remarkable characters of the new species that easily distinguish it from the two Palawan species. At its type locality, *V. exiguum* was observed to be sympatric with *V. palawanense*.

Due to the paucity of collections and relative lack of study, intraspecific morphological variation within the species of Philippine *Vaccinium* (Salares et al. 2018), as well as sectional boundaries and composition, are imprecisely known (Vander Kloet and Dickinson 2009; Fritsch et al. 2020). A detailed monographic study of this group is warranted.

## Supplementary Material

XML Treatment for Vaccinium exiguum
